# Smoking Cessation Using Wearable Sensors: Protocol for a Microrandomized Trial

**DOI:** 10.2196/22877

**Published:** 2021-02-24

**Authors:** Laura M Hernandez, David W Wetter, Santosh Kumar, Steven K Sutton, Christine Vinci

**Affiliations:** 1 Moffitt Cancer Center Tampa, FL United States; 2 Department of Population Health Sciences University of Utah Salt Lake City, UT United States; 3 Department of Computer Science University of Memphis Memphis, TN United States

**Keywords:** mHealth, microrandomized trial, smoking cessation, mindfulness, tobacco, mobile phone

## Abstract

**Background:**

Cigarette smoking has numerous health consequences and is the leading cause of morbidity and mortality in the United States. Mindfulness has the ability to enhance resilience to stressors and can strengthen an individual’s ability to deal with discomfort, which may be particularly useful when managing withdrawal and craving to smoke.

**Objective:**

This study aims to evaluate feasibility results from an intervention that provides real-time, real-world mindfulness strategies to a sample of racially and ethnically diverse smokers making a quit attempt.

**Methods:**

This study uses a microrandomized trial design to deliver mindfulness-based strategies in real time to individuals attempting to quit smoking. Data will be collected via wearable sensors, a study smartphone, and questionnaires filled out during the in-person study visits.

**Results:**

Recruitment is complete, and data management is ongoing.

**Conclusions:**

The data collected during this feasibility trial will provide preliminary findings about whether mindfulness strategies delivered in real time are a useful quit smoking aid that warrants additional investigation.

**Trial Registration:**

Clinicaltrials.gov NCT03404596; https://clinicaltrials.gov/ct2/show/NCT03404596

**International Registered Report Identifier (IRRID):**

DERR1-10.2196/22877

## Introduction

### Tobacco Use and Stress

Cigarette smoking has numerous adverse health consequences and is the leading cause of morbidity and mortality in the United States [1]. Determining how to prevent relapse following a quit attempt is key to successful long-term tobacco abstinence. When considering predictors of relapse, the experience of stress, or negative affect, is strongly associated with the risk of relapse [2,3], and high stress is related to an increased likelihood of lapse during a smoking quit attempt [4]. Developing smoking cessation interventions that target stress are needed and may be particularly useful for individuals with low socioeconomic status (SES), given increased exposure to chronic stressors and other negative life events (eg, discrimination and violence) [5-8].

### Mindfulness as a Quit Smoking Aid

One potential skill that could aid individuals in successful cessation, by enhancing resiliency to stressors, is the cultivation of mindfulness. Mindfulness is a multifaceted construct and is often described as the ability to pay attention to the present moment, with purpose and without judgment [9]. When applied to making a behavior change, such as quitting smoking, mindfulness is hypothesized to decrease automaticity, such that an individual chooses how to respond in a given situation (vs automatically reaching for a cigarette, for instance) [10-13]. Mindfulness is also posited to enhance an individual’s ability to be present with discomfort, which is particularly relevant when managing cravings and symptoms of withdrawal [14]. Mindfulness may be particularly useful for those experiencing high levels of stress, as it teaches skills that are applicable to a host of experiences (eg, managing physical and emotional distress) and is not specific to only changing the targeted behavior of smoking.

Mindfulness-based interventions have been effective in reducing smoking [15-18] and have results comparable with those of other empirically supported treatments (ie, cognitive behavioral therapy) [18]. Most importantly, mindfulness is linked to underlying mechanisms associated with abstinence, such as decreasing negative affect [19-23], increasing positive affect [20,23,24], increasing self-efficacy [25-28], and lowering craving [29-32]. Nonetheless, studies that have examined mindfulness for smoking cessation have typically used the traditional format of mindfulness interventions (8 weekly group sessions lasting 2-2.5 hours). Although the results have been promising, this format is time- and resource-intensive and may discourage engagement, particularly among low SES populations. It is very likely that aspects of mindfulness, when delivered at key moments during the quit smoking process, can also be beneficial.

### Microrandomized Trials

This study uses a microrandomized trial (MRT) design to deliver mindfulness strategies (referred to as ecological momentary interventions [EMIs]) to participants on a smartphone at key moments during a quit attempt. Using MRTs is an innovative way to investigate whether certain intervention components, when delivered at certain times, impact hypothesized mechanisms and behavior [33]. We are specifically interested in the impact of these EMIs in relation to negative affect and lapse [4,6,34-37]. Thus, during a quit attempt, moments will be randomized to either receive a strategy or not, based on (1) times of low and high negative affect and (2) smoking status (lapse and no lapse). Negative affect and smoking status will be detected unobtrusively via wearable wireless sensors, and EMIs will be delivered via smartphones.

### Study Aims

This feasibility study will evaluate the provision of real-time, real-world mindfulness intervention strategies among a sample of smokers making a quit attempt. Outcomes are consistent with those evaluated in other feasibility studies [38,39]. Our feasibility outcomes will include participant retention, adherence, and score on the System Usability Scale. We will also measure acceptability outcomes that will include response to the Client Satisfaction Questionnaire and mindfulness strategies. As an exploratory outcome due to the small sample size, we hypothesize that providing mindfulness strategies in real time will be associated with lower negative affect, greater self-regulatory capacity, and a reduced likelihood of lapsing.

### Goal of the Paper

This paper will describe the protocol for Time2QuitMindfully, which is a study that will evaluate the feasibility of an MRT to deliver mindfulness strategies at key moments in the real world during a quit smoking attempt.

## Methods

### Recruitment

All recruitment and study procedures have been approved by Moffitt Cancer Center’s institutional review board. This study aims to recruit 24 participants who are interested in quitting smoking. To be eligible, participants must be aged 18 years or older; smoke an average of at least three cigarettes per day over the past year; have a carbon monoxide (CO) reading of at least six parts per million (ppm); are motivated to quit within the next 30 days; have a valid home address; have a functioning telephone number; and can speak, read, and write in English. Individuals will be ineligible if they have a history of contraindications for using the nicotine patch unless a doctor’s note is provided, endorse current psychosis, have a pacemaker or implanted device similar to a pacemaker, use smoking products other than cigarettes and e-cigarettes, are pregnant or trying to become pregnant or are breastfeeding, physically unable to wear the equipment and provide good readings of physiological measures, currently trying to quit smoking, involved in a quit smoking program, using tobacco cessation medications, have another household member enrolled in the study, or have no prior experience using a smartphone.

### In-Person Procedures

#### Recruitment, Orientation, and Study Visits

Participants will be recruited from the community via a variety of methods: community flyers, web-based advertisements, and prior study databases containing the contact information of participants who agreed to be contacted about future research opportunities. Interested individuals will be screened over the phone to assess their eligibility. If eligible, individuals will be given more details about what the study entails and will be invited to schedule an in-person orientation session, which will take place either in a group or individually. At orientation, the informed consent process to collect eligibility data will be conducted. Next, individuals’ CO will be measured to assess smoking status, a pregnancy test will be performed to rule out pregnancy, and the Mini International Neuropsychiatric Interview [40] will be conducted to rule out psychosis. If eligible, they will be shown a PowerPoint presentation to learn more about the study and to ask questions. After the PowerPoint presentation, participants who are interested in participating will be scheduled to attend the study visits.

**Figure 1:**
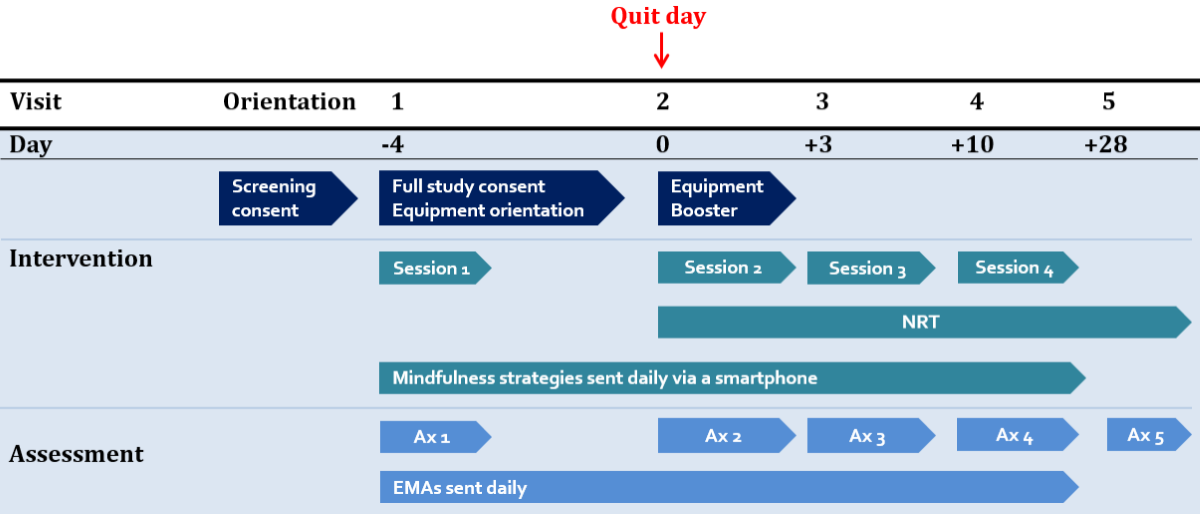
Study timeline. Ax: assessment; EMA: ecological momentary assessment; NRT: nicotine replacement therapy.

The visit schedule is as follows: visit 1 is scheduled to occur 4 days before the quit day, visit 2 is on the quit day, visit 3 occurs 3 days after the quit day, and visit 4 occurs 7 days later. At 28 days after quit day, participants will return for a follow-up visit at visit 5. Figure 1 shows an overview of the timeline of the study sessions. Most sessions will last 1-3 hours, with visit 1 being the longest session at 3 hours.

At the beginning of visit 1, participants will complete the informed consent process for study participation, which involves the experimenter explaining the purpose of the study, why they are being asked to participate, that participation is voluntary, what will happen during the study period, benefits, risks, and confidentiality. All study information presented at orientation will be revisited at visit 1, before having participants sign the informed consent document. After consent is complete, participants will be asked how many cigarettes they currently smoke per day to determine the correct dosage for nicotine replacement therapy (NRT) administration (details of patch administration described further below). CO, height, and weight will be measured, and participants will complete a battery of baseline questionnaires. Questionnaires measure constructs such as mindfulness, stress, anxiety, tobacco history, financial strain, and previous use of technology. The full list of questionnaires for each visit is presented in Table 1.

In the second portion of visit 1, participants will be trained in detail on how to use the study equipment. First, all pieces of equipment (2 wrist sensors, 1 chest box, 1 chest band, 2 types of electrodes, and 1 study phone) will be laid out on a table, and each piece of equipment and their purpose is to be explained in detail by the experimenter. The experimenter will then assemble the equipment on a mannequin one by one to demonstrate how equipment should be worn and will answer any questions along the way. Participants will be presented with their own set of equipment and asked to put it on, and the experimenter will leave the room. The experimenter will then return and check for correctness. After this is completed, there will be a discussion with the participants to ensure they will be wearing the sensors every day (at least 8-12 hours), and to set up a start and end time for each day on the phone.

The experimenter will then go through a participant packet that explains how to put on the equipment and navigate the study apps’ features, such as privacy mode (participants can choose if they do not want data collected for a period of time). The packet also details how to identify data quality, access the troubleshooting features, take off the equipment, end data collection if needed, and charge the equipment overnight. General information on how to use a smartphone and tackle common issues, such as poor data quality, will also be addressed. Before the session is over, the experimenter will ensure that all data quality collected on the phone is optimal and that the equipment is functioning as expected. Participants will also be provided phone numbers to contact if they experience any equipment issues. Equipment training will take approximately 1 hour to complete. At the end of the visit, participants will receive brief counseling and will be given instructions on wearing the patches for their quit day (visit 2). More details on the intervention are provided below.

During visits 2, 3, and 4, the following procedures will occur: assessment of cold or flu-like symptoms since the last visit (to account for if needed when analyzing physiological data), CO collection, administration of questionnaires, equipment check, presentation of technology boosters and provision of additional electrodes, brief counseling, and patch administration. Participants are to return their assigned equipment at visit 4, after 2 weeks of use. A follow-up visit will occur 28 days after quit day (visit 5), where tobacco use will be assessed, questionnaires completed, patches administered, and quit smoking resources provided.

**Table 1:** Study measures overview.

Measure			Visit 1, baseline (−4)		Visit 2, quit day (0)		Visit 3, (+3)		Visit 4, end of Tx^a^ (+10)		Visit 5, follow-up (+28)
Demographics			X^b^		—^c^		—		—		—
**Agency and acculturation**											
	Self-Efficacy Scale-Smoking [41]	X		X		X		X		X	
	Financial Strain Measure [42]	X		—		—		—		—	
	Subjective Social Status [43]	X		—		—		X		X	
**Affect, stress, alcohol, and mental health**											
	Positive and Negative Affect Scale [44]	X		X		X		X		X	
	Perceived Stress Scale [45]	X		X		X		X		X	
	Alcohol Use Disorders Identification Test [46]	X		—		—		X		X	
	Patient Health Questionnaire-Alcohol [47]	X		—		—		X		X	
	Center for Epidemiologic Studies Depression Scale [48]	X		—		—		X		X	
	Generalized Anxiety Disorder-7 [49]	X		—		—		X		X	
	Distress Tolerance Scale [50]	X		—		—		X		X	
	Difficulties in Emotion Regulation Scale-Short Form [51]	X		—		—		X		X	
**Mindfulness and personal resources**											
	Shift and Persist [52]	X		—		—		X		X	
	Five Facet Mindfulness Questionnaire [53]	X		—		—		X		X	
	Mindful Attention Awareness Scale [54]	X		—		—		X		X	
	Mindfulness Self-efficacy [28]	X		—		—		—		X	
	Toronto Mindfulness Scale [55]	X		X		X		X		X	
**Smoking**											
	Wisconsin Smoking Withdrawal Scale [56]	X		X		X		X		X	
	Brief Wisconsin Inventory of Smoking Dependence Motives [57]	X		—		—		X		X	
**Smoking status and biochemical verification**											
	Tobacco history	X		—		—		—		X	
	CO^d^ reading	X		X		X		X		X	
	TLFB^e^	—		X		X		X		X	
	Tobacco Abstinence Questionnaire	—		X		X		X		X	
**Other**											
	Experience with technology survey	X		—		—		—		—	
	Experience with mindfulness survey	X		—		—		—		—	
	System Usability Scale [58]	—		—		—		X		—	
	Feasibility of technology survey	—		—		—		X		—	
	Mindfulness strategies feedback survey	—		—		—		X		X	
	Client Satisfaction Questionnaire [59]	—		—		—		X		—	

^a^TX: treatment.

^b^Procedure occurred at the indicated visit.

^c^Procedure did not occur at indicated visit.

^d^CO: carbon monoxide.

^e^TLFB: timeline follow back.

#### Check-Ins and Troubleshooting With Study Staff

During the 2 weeks of participants wearing the data collection equipment (visits 1-4), the study staff will monitor equipment use via a dashboard. Staff members will be able to see the quality of participant data (eg, wrist sensors, electrocardiography, and respiration) as well as whether participants have been receiving and completing surveys and strategies on the study phone. To troubleshoot and resolve issues, participants will be contacted if poor or no data appear in the system. Participants will be given phone numbers to contact if they experience issues during business hours and weekends.

#### Compensation and Incentives

Participants will be compensated at the end of each visit for completing questionnaires and to cover the costs of transportation, childcare, etc. At orientation, the participants will be compensated with US $10 for attending. At visits 1-3, participants earn US $30 in each session and US $50 at visits 4 and 5. Participants can also earn bonuses throughout the study by answering survey questions administered daily via the study phone. Participants will be compensated with US $1.25 for completing each phone survey if they have worn the on-body sensors at least 60% of the time since the last phone survey or US $0.50 for completing each phone survey if they have not worn the sensors at least 60% of the time. This pay schedule was developed to encourage continuous use of the equipment [60]. Depending on how often participants wear the on-body sensors and complete the surveys over the 14 days, they can earn US $0-$11.25 per day, for a total of US $0-$157.50 over the entire study, in bonuses.

### Equipment

**Figure 2:**
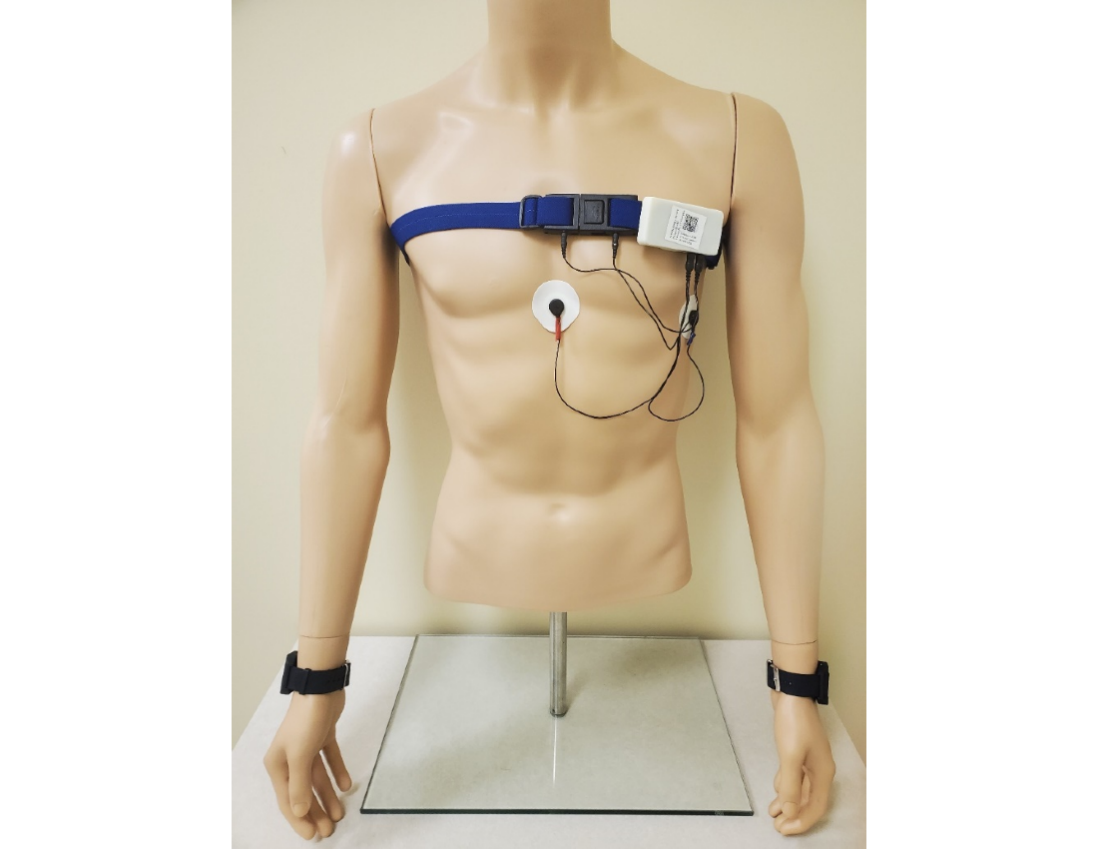
Autosense equipment.

At visit 1, participants will be given a set of equipment to wear for 2 weeks. The equipment suite is called AutoSense [61] and consists of a Samsung Galaxy S5, 2 wrist sensors that look and feel similar to watches or Fitbits, a chest box, and a chest band (Figure 2 shows an image of the equipment) [62,63]. The study phone will have the mCerebrum software [64] that restricts the participant to only accessing the study app when using the phone; this procedure is in place to reduce the use of the phone battery. When the participant turns on the phone, they will see a home page with the study’s contact information and app icon that will lead them directly to the app. The phones will upload data automatically to the Cerebral Cortex cloud [65] and include battery packs that make all-day use possible.

Participants will be given 2 wrist bands that are labeled with either an L or R on the straps, so that they can be placed on the appropriate wrist for data collection. The chest strap can be adjusted to accommodate a participant’s size, and participants will be instructed to wear the band around their chest right under the armpits, under their clothes. The strap has 2 ports where the chest box can be connected. The chest box is a small white box with 2 sets of wires clipped onto the chest band. One set of wires will connect to ports located on the chest band. The other set of wires will connect to the electrodes that are to be placed in the middle of the chest, right below the sternum, and on the left-hand side of the ribcage. Participants will be given 2 types of electrodes and instructed to wear what they are most comfortable with. Participants will also be provided a charging station and a draw string bag to transport their set of equipment and other materials to and from the laboratory.

#### Ecological Momentary Assessments/Surveys on Phone

Ecological momentary assessments (EMAs) will be delivered randomly within prespecified time blocks as well as after a subset of EMIs during the 2-week period. All methodologies for randomly allocating EMAs were preprogrammed using a random number generator function. Randomization also considers what has already been sent historically during that day and is based on certain conditions, as described below. Random EMAs are delivered within 3- to 4-hour blocks throughout the day. Thus, participants will receive no more than 1 survey per 4-hour block to limit the participant burden (up to 3 random EMAs per day). The system is also configured to deploy EMAs after EMIs (described below), 50% of the time. For an EMA to be delivered, certain conditions must be met: time since the last delivered EMI followed by EMA must be more than 15 min, data quality must be classified as good (ie, sensors are attached correctly to the user and the sensors are communicating correctly with the smartphone; red, yellow, and green indicators will be visible to participants so they can fix the equipment if needed. This allows for the sensor data collected in the vicinity of the moment of EMA to be available for analysis), and the participant cannot be driving or engaging in physical activity (to increase the likelihood of responding) [66].

When it is time to take a survey, participants will receive a notification on their phone. Participants will have the option of completing it, delaying it for 5 min, or canceling it altogether. EMAs take an average of 3-5 minutes to complete and assess the following broad areas: mindfulness, affect, motivation, urge to smoke, expectancies, self-efficacy, smoking behaviors, patch use, social setting, alcohol use, discrimination, stressors, and general emotional support. An item assessing whether participants have been using the mindfulness strategies, without the aid of their smartphone, will also be presented.

### Intervention Components

#### EMIs

The use of technology in this study allows participants to receive interventions in real time via the study smartphone in their natural environment. The study app on the phone has been configured to deploy specific interventions triggered by smoking status or the level of negative affect. Data are continuously collected via the sensors. Here, we describe the content of the EMIs, followed by the delivery procedure.

**Figure 3:**
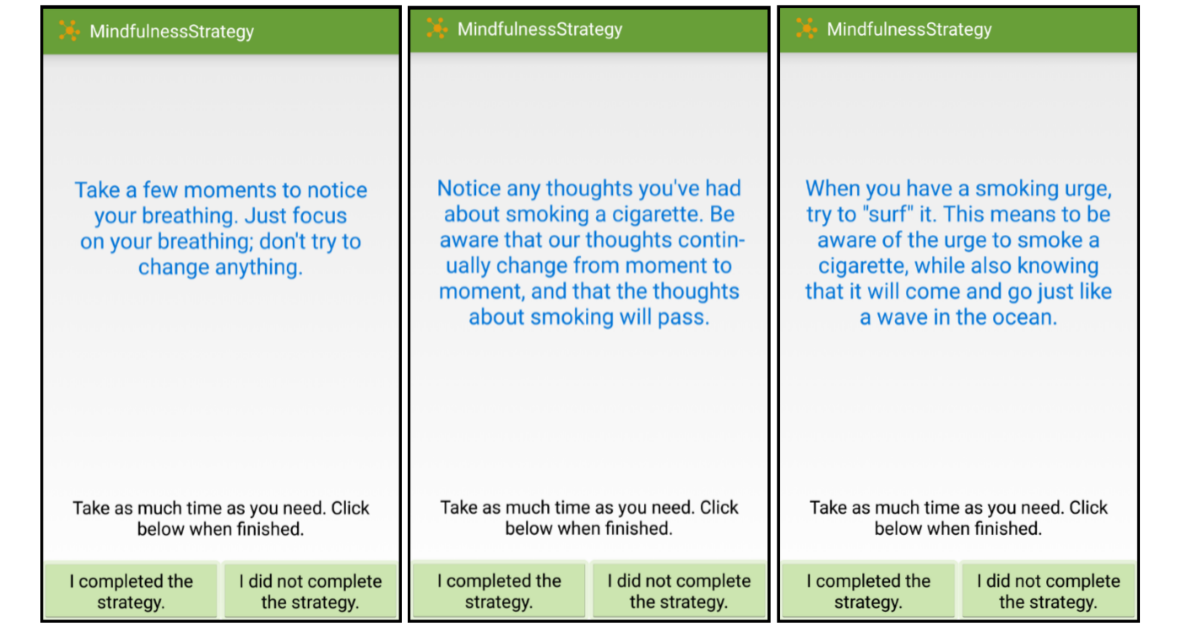
Mindfulness strategies.

Active strategies include prompts to engage in mindfulness-based strategies and read motivational messages throughout the day. The majority of messages will be mindfulness strategies (n=76), which fall into the following topic areas: breath (eg, Turn your attention to your breathing. Notice where you feel your breathing most in your body), thoughts (eg, Observe any thoughts you are having right now. Watch them go by, just as leaves float down a stream of water—they just come and go), sensations (eg, Shift your attention to what you see around you. Be aware of the colors, shapes, textures, and shadows for the next several moments), acceptance/nonjudging (eg, Try to let go of labeling things as good or bad. Be present with what is happening and suspend judgment), and craving (eg, Whenever you have a craving to smoke, think of the craving like a wave in the ocean—it will come and go over time). Examples of motivational messages (n=40) are: “Quitting smoking may be uncomfortable at times, but it will get easier! Your cravings will become less frequent and less intense the longer you go without smoking. Don’t give up!” Figure 3 provides screenshots of the example strategies shown to the participants.

Strategy content is personalized in various ways to the participant. First, it is tailored to whether the participant is in the prequit or postquit portion of the study. For example, a prequit strategy would be “You’re doing a great job preparing to quit smoking!”, whereas a postquit strategy would be “Quitting is a process and takes time. Each hour you go without a cigarette is a step toward your freedom from nicotine. You’re doing great!” Second, strategies in the postquit phase are based on whether a participant has experienced a smoking lapse. An example of a lapse strategy is “Draw your attention to any thoughts you may be having about smoking. Remind yourself that you can get right back on track if you slipped off.” Third, participants rate how much they like a given strategy on a 5-point scale. If they rate it as one star (the lowest rating), they will not see that strategy again.

**Figure 4:**
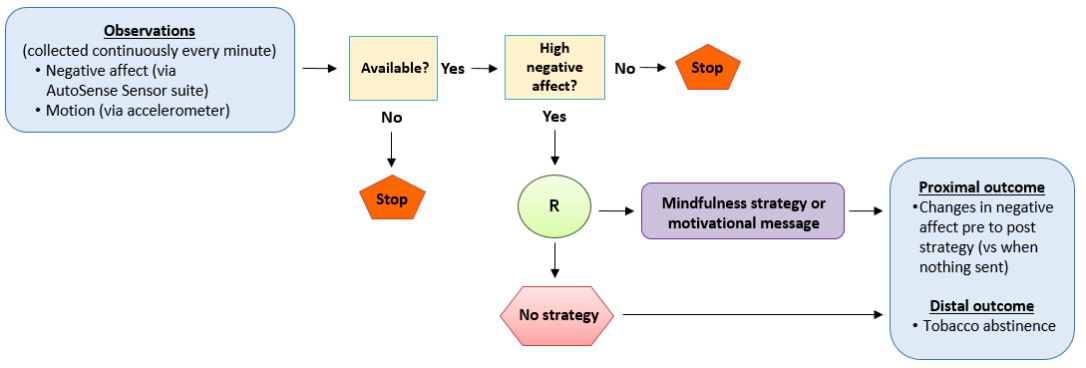
Randomization process based on the detection of high negative affect (low negative affect, lapse, and no lapse are not represented here for simplicity purposes). R: randomization.

The day is separated into six 2-hour blocks for the delivery of EMIs. The decision point for randomization (ie, to send an active strategy or not) is based on smoking status and negative affect, both of which are being continuously monitored every minute while the equipment is being worn. For high negative affect, the decision to randomize is made as soon as a high negative affect event is detected within a 2-hour block and the participant is available. For lapse (ie, the detection of smoking), the decision to randomize is also made as soon as smoking is detected within the 2-hour block and the participant is available. Within a 2-hour block, this process is limited to one randomization occasion for high negative affect and one randomization occasion for lapse. For low negative affect, the decision point to randomize is randomly chosen at the start of the block, and if available when that time arrives, randomization occurs. If the participant is not available, another time is randomly chosen within that time block, and when that time arrives, assuming the participant is available, randomization occurs. This pattern is repeated until the end of the block. The process for randomization for no lapse is identical to the process of low negative affect, as previously described. Within a 2-hour block, randomization is limited to one occasion for low negative affect and one occasion for no lapse. In summary, up to 4 randomizations can occur within a 2-hour block (high negative affect, lapse, low negative affect, and no lapse). Moments will be randomized to receive a strategy or not in a 1:1 manner, and factors to ensure this 1:1 randomization will be included in the algorithm (ie, inclusion of historical data of what has already been triggered that day). Figure 4 shows the process of randomization based on the detection of high negative affect (low negative affect, lapse, and no lapse processes are not included in the figure for simplicity purposes, but their procedures mimic what is shown for high negative affect).

When considering low/high negative affect and lapse/no-lapse occasions, participants can receive up to 2 active strategies within a block (ie, they receive a mindfulness strategy or motivational message). Thus, participants can receive up to 12 active strategies per day. Other conditions are also considered for the delivery of the strategies. These include time since last EMA being at least 15 min, time since last EMI being at least 30 min, good quality data detected via sensors, no driving, phone battery level at least 10%, and no engagement in physical activity. Given the above conditions for the delivery of EMIs, we do not anticipate individuals receiving more than 6 per day, and they may even receive fewer than 6. For instance, if someone does not lapse, then no strategies are sent for that purpose. AutoSense will not send messages when it detects driving, so no messages are sent during those times.

When active strategies are sent to participants, they will receive a notification on the phone, allowing them to know that a strategy is available. They will have the option of accepting it, delaying it for 5 min, or canceling it altogether. When participants agree, they will have the option of receiving a new strategy if they do not like the first one that appears on the phone. Participants will then let the app know whether they completed the strategy, and if they indicate *yes*, they will be asked to rate how much they liked the strategy on a scale of 1 to 5 stars. If they select 1 star, then this strategy will not be shown again to the participant.

#### On-Demand Mindfulness Content

In addition to the strategies and surveys that will be sent to a participant’s phone, participants will also have the opportunity to practice strategies on their own (ie, user-initiated). There is a mindfulness strategy button within the app that can be clicked at any time by the participant to receive a strategy. Although participants will be choosing to engage in a strategy as opposed to being sent one, the process will be similar to that described above (eg, rating the strategy). Participants will be informed at visit 1 that they can click on this button at any time. The phone also has a Mindfulness Frequently Asked Questions (FAQ) button that participants can click at any time to learn more about mindfulness. This feature contains 13 common questions about mindfulness, such as *What is mindfulness?* and *How does being aware of my thoughts relate to me quitting smoking?*

#### In-Person Counseling

During visits 1-4, participants will receive brief counseling (20-30 min) at the end of their sessions. Counseling will be consistent with the Treating Tobacco Dependence Guidelines [67] and will also incorporate a brief mindfulness component. At visit 1, participants will be provided with the National Cancer Institute’s Clearing the Air Booklet [68] as well as instructions on how to use the nicotine patch. They will also be given a single-page handout on mindfulness (eg, definition of mindfulness and why we suggest using mindfulness to help quit smoking). In visits 1-3, participants will listen to a 10-minute audio recording of a mindfulness meditation. At visits 1 (prequit) and 3 (postquit), participants will listen to a recording on urge surfing; at visit 2 (quit day), an audio recording of guided breath meditation will be used. Following these meditations, the counselor will ask questions such as *What did you notice while listening to the recording?* and *How do you think what you just heard is related to quitting smoking?*

#### NRT

Participants will receive 6 weeks’ worth of 14 mg or 21 mg nicotine patches throughout the duration of the study. At visit 1, participants will receive patches and instructions on how to use them. Participants will be instructed to begin wearing the patch at the beginning of the day of their visit 2 (quit day). If a participant smokes 10 or less cigarettes per day, they will be given 14 mg patches. If the participant smokes more than 10 cigarettes per day, they will be given 21 mg patches. At visit 5 (follow-up), participants will receive 2 weeks’ worth of the dosage that they have been using for the last 28 days (totaling 6 weeks of patches) and will be given additional guidance on how to continue the patch regimen. At the end of the last visit, participants will also be given recommendations on how to obtain nicotine patches from community sources as well as additional quit smoking and counseling referrals, if needed.

### Analyses

#### Feasibility Outcomes

Our measures of feasibility and associated benchmarks of success include the following: retention (≥75% through follow-up), adherence (eg, expect ≥70% of participants to wear the equipment the majority of the 14 days; of those who wore the equipment the majority of the 14 days, ≥70% completion of EMAs; ≥60% of strategies completed), and a score of 68 or higher on the System Usability Scale. Acceptability will be primarily driven by scores on the Client Satisfaction Questionnaire (≥3), followed by responses to the mindfulness strategies.

#### Exploratory Outcomes: Sensor-Based Collection of Negative Affect, Self-Regulatory Capacity, and Smoking Status

Wearable sensors are configured to detect negative affect, self-regulatory capacity, and smoking status, to deliver and ultimately collect outcome data on relevant intervention content to the study phone. The experience of negative affect is detected through a machine learning model applied to continuous sensor-based measurements based on electrocardiogram (ECG) and respiration data [62]. Respiration data are measured via inductive plethysmography (RIP), which is the contraction of the chest and abdominal wall, as collected by the chest band [62]. ECGs are measured via 2 electrodes connected to a chest box unit. Probability of experiencing negative affect is derived from these measures, which will be used as our indicator of negative affect; additional detail can be found in the study by Hovsepian et al [62]. Self-regulatory capacity will be captured via heart rate variability, which is also captured by the ECG data.

Smoking episodes are detected via the *puffMarker* model, a machine learning algorithm that uses respiration data to detect deep inhalation and long exhalation and wrist movement patterns to detect hand-to-mouth gestures [63]. Inhalation and exhalation data are collected via RIP sensors, whereas specific wrist movements indicative of smoking are collected by wrist bands with a 3-axis accelerometer and a 3-axis gyroscope [63]. All of the data collected by the sensors are continuously measured and wirelessly streamed to the study phones and then sent to the cloud for analysis [63].

#### Tobacco Abstinence

Tobacco abstinence will be defined as biochemically confirmed 7-day point prevalence, which is the self-report of no smoking in the past 7 days combined with a CO reading of <6 ppm. Self-reports of smoking status will be collected via the timeline follow back at visits 2-5.

#### Analytic Plan

Descriptive statistics (eg, means and percentages) will be derived for feasibility and acceptability outcomes. Given that this is a feasibility study, sensor-driven analyses will be exploratory with the goal of providing preliminary results. The unit of analysis is the pre/postengagement data pairs comparing the average negative affect, self-regulatory capacity, and lapse counts within a prespecified time frame (eg, negative affect and self-regulatory capacity in the 30 minutes before and after randomization and lapse status in the time period between randomization of EMIs). These data will be analyzed using a generalized linear mixed model with proper adjustment for the within-subject correlation. The model can also include indicators for the category of mindfulness strategy. Each variable pair (negative affect, self-regulatory capacity, and smoking) will be analyzed separately in a pre/postengagement comparison to determine whether the mindfulness intervention creates any changes in these variables when compared with receiving no strategy at all. Examples of potential time-invariant covariates that will be included in the model are gender, age, education level, baseline trait mindfulness, and nicotine dependence. Time-varying covariates to be considered in the models include smoking status and use of the *on-demand* mindfulness content. Given that MRTs require unique considerations regarding data analysis (eg, participant availability and changes in the use of the intervention over time), we will incorporate the most recent recommendations in the literature when examining the data [69,70].

## Results

Recruitment is complete, and data management is ongoing. This study was funded in February 2017 and received IRB approval in February 2017; data collection occurred from January 2019 to November 2020 (note that some of recruitment occurred during COVID-19). As of the submission of this manuscript, 43 participants consented to participate in the full study.

## Discussion

This manuscript describes a study that aims to recruit individuals who are motivated to quit smoking (N=24), with the overall goal of testing whether a 2-week mindfulness-based MRT is feasible as a quit smoking treatment. All participants will receive brief mindfulness-based strategies in real time, NRT, and 4 brief smoking cessation counseling sessions. Participants will be asked to wear AutoSense equipment that gathers physiological data and to carry a study smartphone that will deliver EMAs and EMIs to them during the initial weeks of their quit attempt. Participants will attend 1 follow-up visit after treatment.

Limitations of this study, along with the next steps, should be noted. First, this study will recruit a small sample size consistent with feasibility studies [38,71], which will limit any conclusions that can be made. Second, this study requires participants to wear equipment that could be burdensome or complicated to navigate, which may impact compliance with the intervention. Third, the inclusion of in-person brief counseling and NRT limits our ability to determine the impact of the mobile health (mHealth)-only aspects of the intervention. Nonetheless, we thought it is best to provide basic evidence-based cessation treatment to participants (pharmacotherapy plus brief counseling), given the novelty of the mHealth intervention component. Future research may choose to deliver and evaluate a remote-based treatment (ie, mail-out NRT and conduct phone counseling) or to provide an mHealth-only treatment with the entire smoking cessation intervention delivered via a smartphone and sensors. On the basis of the findings of this study, the next steps should include a large-scale randomized clinical trial to compare this treatment with a usual care/control condition to ultimately determine treatment efficacy.

This study will provide initial data on whether brief mindfulness strategies, delivered in real time at key moments during a quit smoking attempt, are a useful quit smoking aid. This project is highly innovative because (1) to date, no studies have examined these constructs via real-world, real-time data among smokers and (2) findings can directly inform treatment development to specify how mindfulness impacts underlying mechanisms, leading to the reduction of tobacco-related health disparities.

## Abbreviations

CO: carbon monoxide
ECG: electrocardiogram
EMA: ecological momentary assessment
EMI: ecological momentary intervention
mHealth: mobile health
MRT: microrandomized trial
NRT: nicotine replacement therapy
ppm: parts per million
RIP: inductive plethysmography
SES: socioeconomic status
